# Betaine Modulates Rumen Archaeal Community and Functioning during Heat and Osmotic Stress Conditions *In Vitro*

**DOI:** 10.1155/2020/8875773

**Published:** 2020-10-22

**Authors:** Mubarik Mahmood, Ratchaneewan Khiaosa-ard, Qendrim Zebeli, Renée M. Petri

**Affiliations:** ^1^Institute of Animal Nutrition and Functional Plant Compounds, Department for Farm Animals and Veterinary Public Health, University of Veterinary Medicine Vienna, 1210 Vienna, Austria; ^2^Section of Animal Nutrition, Department of Animal Sciences, University of Veterinary and Animal Sciences, Lahore, Subcampus Jhang, 12 km Chiniot Road, Jhang 35200, Pakistan; ^3^Agriculture and Agri-Food Canada, Sherbrooke Research and Development Centre, 2000 College Street, Sherbrooke, QC, J1M 1Z7, Canada

## Abstract

Rumen archaea play an important role in scavenging ruminal hydrogen (H_2_) and thus facilitate rumen fermentation. They require optimum temperature and osmolality for their growth and metabolism; however, a number of external factors may put archaea under heat and osmotic stress. Betaine is an osmolyte, molecular chaperone, and antioxidant; therefore, it bears potential to combat against these stressors. In this *in vitro* study, three betaine levels, namely, 0 (control), 51 (low), and 286 (high) ppm, were used. Each of these was subjected to two temperatures (39.5 and 42°C) and two osmolality conditions (295 and 420 mOsmol kg^−1^) with *n* = 6 per treatment. Sequencing analyses of the solid phase (which use solid materials containing primarily fibrous materials of low-density feed particles) and the liquid phase (rumen fermenter liquid) using 16S rRNA revealed that more than 99.8% of the ruminal archaea in fermenters belong to the phylum *Euryarchaeota*. At the genus level, *Methanobrevibacter* was the most prevalent in both phases, and *Methanosaeta* was only detected in the liquid phase. The genera *Methanobrevibacter* and *Methanobacterium* both showed a positive correlation with methane (CH_4_) formation in the liquid and solid phases, respectively (*P* < 0.05). Heat stress increased the relative abundance of genus *Methanimicrococcus* at the expense of candidate archaeal genus Vadin CA11 (*P* < 0.05). In the solid phase, osmotic stress significantly reduced the Shannon and Simpson indices of diversity, and relative abundance was higher for *Methanobrevibacter* at the expense of *Methanimicrococcus*. In the liquid phase, osmotic stress increased not only the abundance-based coverage estimator (ACE) and singles parameters of diversity but also the relative abundances of *Methanosphaera* and *Methanobacterium*. The overall decrease in all gas parameters and estimated metabolic hydrogen ([2H]) utilization was observed during osmotic stress conditions (*P* < 0.05). Betaine enhanced the diversity of solid phase archaea as indicated by the increase in ACE and singles during heat stress, and only a high dose improved all diversity parameters in the liquid phase during osmotic stress (*P* < 0.05). Thus, betaine alleviates the effects of heat stress and osmotic stress on the archaea community.

## 1. Introduction

Betaine or trimethyl glycine is a zwitterionic, compatible, and widely available organic osmolyte, which is either synthesized or picked up by the microbes to equilibrate their ionic balance and cell turgor [1–3]. Betaine also stabilizes the native protein structure of the cell and prevents molecular disintegration, acting as a molecular chaperone and intracellular antioxidant [4, 5]. These characteristics make betaine suitable for stabilizing bacterial cell metabolism under stress conditions. Betaine is found naturally abundant in wheat bran and sugar beet, both animal feed products, and it has been previously shown to reduce the effects of environmental and dietary changes on the gastrointestinal microbiota [3, 6, 7]. Our previous research looking at the impact of betaine under high temperature and increased osmolality conditions on rumen found that the application of betaine *in vitro* resulted in a stabilization of the bacterial population and improved ruminal fermentation [3]. It is reasonable to expect that the presence of betaine could also affect rumen archaea community and functioning. Firstly, as a compound bearing methyl groups, betaine catabolism carried out by ruminal microbes releases trimethylamine that is subsequently used for methanogenesis [8]. In this way, betaine addition could have a direct impact on methylotrophic archaea, the population that is less studied in rumen environments. Furthermore, according to the aforementioned properties, betaine may help to stabilize the community of archaea when they are subjected to stress. Although the unique structure of the archaeal cell membrane enables them to be more stress tolerant than rumen bacteria [9], they still require optimal rumen temperature (38°C to 41°C) and osmolality (260 to 340 mOsmol kg^−1^) for growth and metabolism [10]. Both physicochemical parameters are sensitive to a number of external factors in the rumen of livestock animals including the use of different dietary components, which possess an ability to resiliently change the osmotic pressure as well as rumen temperature [11, 12]. The consequences of such stressors on the rumen archaea population range from cellular dehydration [13, 14], to single-cell death [15], to a complete disruption of fermentation in the rumen ecosystem resulting in ruminal dysbiosis and a reduction in animal production and health [16, 17]. As methanogenic archaea are known for their vital role in keeping the partial pressure of H_2_ low in the rumen [18], stabilizing the archaeal community and their metabolic function under stress would therefore facilitate fermentation in the rumen. Therefore, although they only represent up to 3.3% of the total microbial mass in the reticulorumen of cows, archaea occupy an important ecological position in the biological network of ruminal microbiota [19]. However, the general application of betaine within the rumen has been minimal to date, and therefore, its influence on the rumen archaea population and function remains unknown. Research data is especially lacking with regard to the rumen ecosystem under stressful ruminal conditions.

To address this research gap, the current study was planned with the objective of determining the effects of betaine on relative abundance and community diversity of rumen archaea in both solid and liquid phases in the rumen under both heat and osmotic stress conditions, using an *in vitro* rumen simulation technique (Rusitec). We hypothesized that both heat stress and osmotic stress would reduce the diversity and relative abundances of the rumen archaea genera in both the solid and liquid phases of the *in vitro* population, and that the addition of betaine under heat stress and osmotic stress would counteract the decreased diversity by stabilizing the relative abundances of rumen archaea and thereby resulting in methanogenesis.

## 2. Material and Methods

### 2.1. Experimental Design and Treatments

The trial was performed using two Rusitec assemblies described in detail earlier [3]. In brief, each Rusitec assembly contained 6 fermenters placed in a water bath, with each fermenter having an effective volume of 800 mL. A total of 6 experimental runs were conducted resulting in *n* = 6 per individual treatment. Each run consisted of 10 days, with the first 5 days (d1–d5) as an adaptation period followed by the last 5 days (d6–d10) of sampling and measurements. The system reached pH stability after 4 days of incubation (Supplementary Figure 1) in line with a previous study by Mickdam et al. [20]. The experiment was based upon a 2 × 2 × 3 factorial design with 2 temperatures (normal—39.5°C; heat stress—42°C), two osmotic conditions (normal—~295 mOsmol kg^−1^ and pH of 6.6; osmotic stress—~420 mOsmol kg^−1^ and pH of 6.0), and 3 betaine supplementation levels (0 ppm—control; 51 ppm—low; 286 ppm—high). The low and high betaine doses were equivalent in providing 0.03 and 0.2 grams of betaine per day per fermenter, respectively. ActiBeet® L (naturally sourced betaine), containing 40% *v*/*v* betaine (AGRANA Stärke GmbH, Vienna, Austria), was used to prepare the respective betaine doses, and the target pH and osmolality were attained with the use of buffer and diet. The forage portion of the diet contained on dry matter basis grass silage (25% in normal, 20% in osmotic stress), corn silage (15% in normal, 7% in osmotic stress), and second-cut meadow hay (10% in normal, 8% in osmotic stress). The concentrate mixture (43% in normal, 56% in osmotic stress) consisted of barley (21.55%), wheat (21.55%), maize (51.7%), and vitamin and mineral supplements (5.2%), in addition to Rindastar (Schaumann, Germany) protein concentrate which was used as protein source (7% in normal, 9% in osmotic stress) [3]. The chemical compositions of both diets has already been reported in detail in another parallel study [3]. The normal osmolality condition was maintained with the infusion of McDougall's salivary buffer [21] with small modifications along with the use of diet containing 50 : 50 ratio of forage : concentrate. The osmotic stress condition was induced by making adjustments to McDougall's buffer and diet with 35 : 65 ratio of forage : concentrate. The treatments were randomized between runs and fermenters to avoid instrument-specific effects. Before the start of the experiment, diet ingredients except hay were oven dried at 65°C for 48 h and then ground with a Wiley mill (Pulverisette 25/19, Fritsch GmbH, Idar-Oberstein, Germany) to pass through a 6 mm sieve.

### 2.2. Rusitec Procedure

On the first day of each experimental run, rumen fluid and solid digesta (solid materials containing primarily fibrous materials of low-density feed particles) were obtained from three cannulated nonlactating Holstein cows maintained at the VetFarm Kremesberg of Vetmeduni, Vienna and fed second-cut meadow hay. Both rumen fluid and solid digesta were collected while approaching through the opening of the ruminal cannula; the former was obtained with the help of a suction pump attached to a hose, and the latter was acquired manually from the rumen mat. Cows were fed hay and grass silage and were kept according to the Austrian guidelines of animal welfare [22]. Rumen contents from all three cows were pooled together by phase. Rumen fluid was first filtered through four layers of medical gauze (1 mm pore size), and then 600 mL was added to each fermenter with 100 mL of the respective buffer. Solid digesta was subsampled and placed in nylon bags (120 × 65 mm, 150 *μ*m pore size, Fa. Linker Industrie-Technik GmbH, Kassel, Germany), then one filled with pooled solid digesta and one filled with corresponding diet (12 g dry matter (DM)) were added to each vessel. After 24 h, the bag with solid digesta was removed and replaced with a fresh bag of the respective diet prepared by mixing feed ingredients. Artificial saliva was provided continuously to all fermenters using a 12-channel peristaltic pump (model ISM932, Ismatec, IDEX Health & Science GmbH, Wertheim, Germany) at a rate of 393 ± 17 mL per day. Outflow was collected in a glass bottle kept at 1°C, and fermentation gases were collected in the gas-tight bags (TECOBAG 8 L, Tesseraux Container GmbH, Bürstadt, Germany). The addition of new feed was done daily at 0800 h, and outflow and gas measurements were taken simultaneously. First, nitrogen gas was flushed through a vessel for 30 seconds to collect all residual gases in the respective gas bag. Subsequently, the fermenter was opened and total outflow was measured. Then finally, the 48 h incubated feed bag was replaced with a new feed bag. Prior to removal from the experiment, the 48 h incubation feed bags were first rinsed with 40 mL of respective buffer and squeezed to promote the transfer of liquid phase microbes back into the fermenter. After resealing the fermenter, nitrogen gas was flushed for 3 minutes to restore anaerobic conditions, and an empty gas bag was attached. Soon after feeding, 600 *μ*L betaine solution was dosed carefully with a pipette via a small valve at the top of the fermenters. The 0 h concentrations for control, low, and high betaine doses were 0.8 ± 1.2, 56 ± 6, and 314 ± 41 ppm (mean ± SD) [3].

### 2.3. Daily Sampling and Measurements

Incubation fluid from fermenters was collected daily with a syringe through the valve prior to feeding. This fluid was used to determine pH and redox potential using a pH meter (SevenMulti™, Mettler Toledo GmbH, Schwerzenbach, Switzerland) furnished with two electrodes (InLab Expert Pro-ISM for pH and Pt4805-DPA-SC-S8/120 for redox, respectively; Mettler Toledo GmbH, Schwerzenbach, Switzerland). On sampling days (d6–d10), an additional aliquot was taken for short-chain fatty acids (SCFA), and on d10, an additional aliquot was taken for archaea analysis. On d10, the feed bag incubated for 24 h was snap-frozen and preserved at -20°C for archaea analysis. Samples for archaea analysis were snap-frozen in liquid nitrogen and stored at -20°C for further DNA extraction and sequencing analysis. Residual feed bags collected during the sampling period were handwashed by running cold water until the water became clear, and preserved at -20°C for chemical analysis.

### 2.4. Chemical Analysis

Analysis of the composition and volume of the collected gas, chemical analysis of feed and feed residues, and SCFA analysis were performed according to a previous study conducted by Humer et al. [23]. In brief, the composition of fermentation gases was analyzed using an infrared detector machine (ATEX Biogas Monitor Check BM 2000, Ansyco, Karlsruhe, Germany), and the volume of gas was measured by the water replacement method. Feed residues were pooled for the last 5 days, and chemical analyses of feed and feed residues were performed according to the handbook of agricultural analytic and research methodology (VDLUFA) [24]. The difference in the composition of nutrients in feed, before and after incubation, was used for the estimation of apparent nutrient disappearances of dry matter (DM) and organic matter (OM). Methane (CH_4_) production is presented as absolute (mL/d), and relative production is normalized by the apparent nutrient disappearances (mL/g degraded DM or OM). SCFA composition (acetate, propionate, n-butyrate, isobutyrate, n-valerate, isovalerate, and caproate) and concentration for individual samples were determined by gas chromatography (GC) (Fisons GC model 8060 MS DPFC, No. 950713, Rodena, Italy) using a flame-ionization detector and a 15 m × 0.530 mm capillary column (SN US46185178, JW Scientific, Folsom, CA). The detector and injector were maintained at specific temperatures of 190 and 170°C, respectively. Helium was used as a carrier gas sustained at a flow rate of 1 mL/min. Final categorization and evaluation of the chromatogram peaks were completed by Stratos Software (Stratos version 4.5.0.0, Polymer Laboratories, Shropshire, UK).

### 2.5. Calculation of Metabolic Hydrogen ([2H]) Balance

The [2H] balance, as shown in Table 1, was calculated from the stoichiometry of fermentation end products described previously [25]. Accordingly, total [2H] production (mmol/d) was estimated as the sum of [2H] from the daily production of acetate, butyrate, and caproate (mmol/d), and total [2H] utilization (mmol/d) was calculated as the sum of [2H] utilized for the production of propionate, valerate, caproate, and CH_4_ (mmol/d). The volume of CH_4_ was converted to moles using the Ideal Gas Law. Caproate can be synthesized from the condensation of 2 propionyl-CoA that requires the incorporation of 4 moles [2H] per mole of caproate or 2 acetyl-CoA that releases 2 moles [2H] per mole of caproate. Both scenarios were assessed to estimate total [2H] produced and consumed. Utilization of [2H] associated with minor fermentation end products including formate and heptanoate [25] was not considered. The estimated [2H] production and utilization were used to calculate the [2H] gain and % [2H] recovery.

### 2.6. DNA Extraction

Total genomic DNA was extracted from about 800 *μ*L of liquid phase and 0.25 g of solid phase using the DNeasy PowerSoil Kit (Qiagen, Hilden, Germany) following the method described by Bagheri Varzaneh et al. [26] with some modifications. In brief, after adding solution C1 and incubating at 95°C for 5 minutes, the samples were centrifuged and supernatants were collected and put on ice for further processing. 100 *μ*L of 100 mg/mL lysozyme and 10 *μ*L of 2.5 U/mL mutanolysin (Sigma-Aldrich, Vienna, Austria) were added to the pellets and incubated at 37°C for 30 minutes. Afterwards, 21 *μ*L of 18.6 mg/mL proteinase K (Sigma-Aldrich, Vienna, Austria) was added followed by incubation at 37°C for 1 h. Mechanical disruption of the archaeal cells was performed by bead beating using the FastPrep-24 Instrument (MP Biomedicals, Santa Ana, CA, USA) according to previously published procedures [27]. After centrifugation, the supernatant of each sample was added to the previously collected supernatant followed by chemical removal of cell debris and PCR inhibitors by several centrifugation steps. The supernatants were transferred to fresh tubes for column-based isolation of total genomic DNA, and DNA was eluted in 100 *μ*L of C6 buffer. DNA concentration was determined by a Qubit 2.0 Fluorometer (Life Technologies, Carlsbad, CA, USA) using the Qubit double-stranded DNA (dsDNA) HS Assay Kit (Life Technologies) and stored at -20°C until further analysis. In order to improve the archaea population identification, a PCR amplicon approach was used whereby a 25-cycle PCR was performed, using 5 ng template and 100 nM of the primers 344F (5′-ACGGGGYGCAGCAGGCGCGA-3′) and 1041R (5′-GGCCATGCACCWCCTCTC-3′; Moissl-Eichinger, personal communication). The PCR was done in a 20 *μ*L reaction volume including 10 *μ*L of Fast Plus EvaGreen Master Mix with low ROX as reference dye (Biotium, Hayward, CA, USA), 1 *μ*L of each forward and reverse primers, 7 *μ*L DEPC-Treated Water (G-Biosciences, St. Louis, USA), and 1 *μ*L template. All reactions were run in duplicates including a negative control on a 96-well plate (VWR, Vienna, Austria) using a Mx3000P Stratagene PCR System (Agilent Technologies) at the following temperatures: 95°C for 5 minutes for initial denaturation, 95°C for 5 seconds for enzyme activation, 64°C for 30 seconds for annealing, and 72°C for 30 seconds for elongation.

### 2.7. Sequencing and Bioinformatic Analysis

Each amplicon sample (5 ng in 20 *μ*L) was sent for amplicon sequencing using Illumina MiSeq paired-end sequencing technology (Microsynth AG, Balgach, Switzerland). Targeted amplification of the V4 of archaeal 16S rRNA gene was performed using the primer sets 515F (5′-GTGCCAGCMGCCGCGGTAA-3′) and 806R (5′-GGACTACHVGGGTWTCTAAT-3′) [28]. The sequencing procedure was as described by Bagheri Varzaneh et al. [26]. On purified PCR products, libraries were constructed by ligating sequencing adapters and indices (Nextera XT Sample Preparation Kit, Illumina, CA) according to the manufacturer's recommendations. Equimolar amounts of each library were pooled together and sequenced on an Illumina MiSeq Personal Sequencer. The resulting paired ends were stitched together by Microsynth AG (Balach, Switzerland). Data quality control and analyses were performed using the open source Quantitative Insights Into Microbial Ecology (QIIME) pipeline (http://qiime.org/) [29]. Screening for chimeric sequences was done using USEARCH [30], and the resulting cleaned sequences were then aligned and clustered to define operational taxonomic units (OTUs) using Python Nearest Alignment Space Termination (PyNAST) (QIIME) [29] and the SILVA-128 database (v128; accessed November 2018) [31]. The degree of similarity between sequences was defined as 97% to obtain OTU identity at the species level. OTUs which clustered with less than 10 reads were manually removed. For alpha diversity analysis, abundance-based coverage estimator (ACE), Shannon and Simpson index, and singles were used. The singles represent OTUs that appeared only once in the sample. Beta-diversity analysis was performed using weighted Unifrac dissimilarity metrics and the principal coordinate analysis (PCoA) plotting in QIIME with rarefaction at 41,921 sequences, based on the lowest number of sequences in a single sample. The total number of raw sequences prior to quality analysis was 10,317,729. Sequencing data are available in the BioProject SRA database under the accession number PRJNA602990.

### 2.8. Statistics

Statistical analyses were performed using the MIXED Procedure of SAS (version 9.4, SAS Institute Inc., Cary, NC, USA). The statistical model included betaine supplementation, incubation temperature, and osmolality along with their 2-way and 3-way interactions. The variation between experimental runs was considered as a random effect. Relative abundance of the microbial populations was also tested using the above statistical model without the use of repeated measures. Correlation analysis was performed using the CORR procedure to obtain Pearson's correlation coefficients. Mean values reported are least square means ± pooled standard error (SE). Significance was declared at *P* ≤ 0.05 and a tendency of an effect at 0.05 < *P* ≤ 0.10.

## 3. Results

### 3.1. Fermentation Gas Production and Composition as Affected by Incubation Conditions and Treatment

Heat stress minimally affected most of the gas parameters, and only the acetate-associated [2H] production showed a tendency to increase with heat stress (Table 2). Heat stress shifted the utilization of [2H] associated with valerate production at the expense of that of propionate (*P* < 0.05). Osmotic stress significantly suppressed all of the fermentation gas parameters including absolute CO_2_ production (-146 mL/d), absolute CH_4_ production (-38 mL/d), CH_4_/g OM degraded (-5.6 mL), CH_4_/g DM degraded (-5.1 mL), and methane conversion rate (-0.71% gross energy (GE) intake; *P* < 0.0001).

For methanogenesis parameters, no treatment × incubation condition interaction was observed. Production of [2H] in relation to acetate production, and utilization in relation to valerate and CH_4_ production were significantly decreased during osmotic stress (*P* < 0.05), which resulted in a significant decrease in overall [2H] production and utilization. There was no change in [2H] gain when caproate was linked to the propanyl CoA pathway, but percent [2H] recovery was lowered by 7.7-7.9% due to osmotic stress (*P* < 0.05).

Regardless of incubation conditions, in comparison to control, only supplementation with the high dose of betaine produced significantly more absolute total fermentation gas (*P* < 0.0001) and CO_2_ (*P* < 0.0001, Table 2). Absolute CH_4_ production, CH_4_ conversion rate, CH_4_ formation per gram of OM and DM degraded, and CH_4_ percentage of total fermentation gas, all remained significantly higher in both the low and high dose of betaine in comparison to control (*P* < 0.05). CH_4_ formation (per gram of OM and DM degraded) was increased by 61 and 95% due to the addition of betaine in the low and high dose, respectively, and an increase in CH_4_ percentage of total fermentation gas only for the high dose was at the expense of CO_2_ (*P* < 0.0001). Overall [2H] production and utilization were enhanced by each level of betaine as compared to control (*P* < 0.05). An increase in [2H] production was seen with an association with acetate (+3.5 low dose, +5.8 high dose) and an increase in [2H] utilization with the formation of CH_4_ (+1.5 low dose, +6.9 high dose, *P* < 0.05). Thus, absolute [2H] gain remained unaffected, but anyway, percent [2H] recovery was raised especially with the high dose in comparison to control through both pathways (*P* = 0.001).

### 3.2. Archaea Diversity as Affected by Incubation Conditions and Treatment

**Figure 1:**
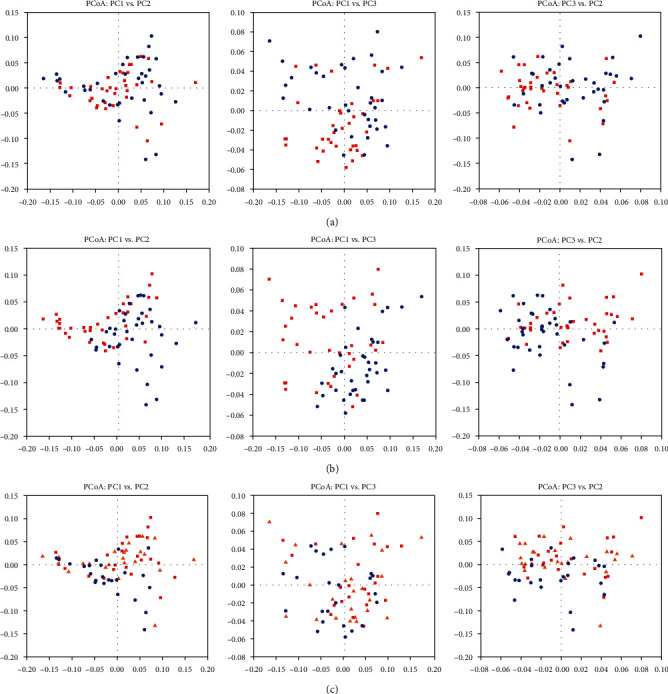
Principle coordinate analysis (PCoA) for beta-diversity of ruminal archaea in the liquid phase of the incubation content of fermenters, under the effects of (a) temperature (red squares: 39.5°C; blue circles: 42°C), (b) osmolality^1^ (blue circles: normal; red squares: osmotic stress), and (c) betaine supplementation^2^ (red squares: control; orange triangles: low; blue circles: high). The first 3 components were plotted and in total principle component explained 69.74% of the total variation (PC1 = 43.07, PC2 = 16.45, and PC3 = 10.22%, respectively). ^1^Normal osmolality ~ 295 mOsmol kg^−1^; hyperosmolality ~ 420 mOsmol kg^−1^. ^2^Betaine levels: control (0), low (51), and high (286) ppm.

**Figure 2:**
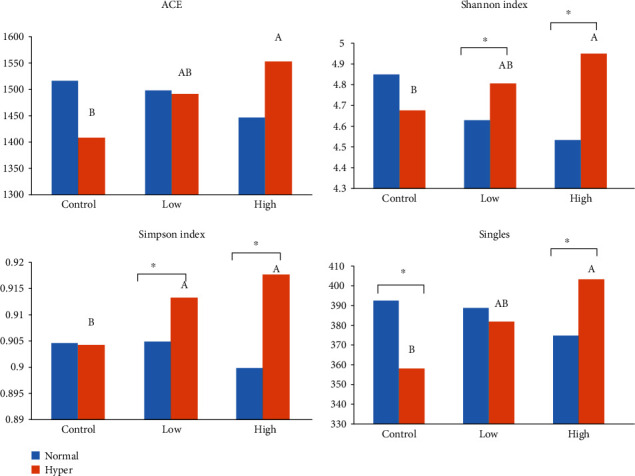
Diversity parameters of ruminal archaea in liquid phase as affected by betaine addition^1^ and osmolality^2^. Different superscripts on hyperosmolality bars represent significant difference (*P* < 0.05). ^∗^*P* < 0.05. ^1^Betaine levels: control (0), low (51), and high (286) ppm. ^2^Normal osmolality ~ 295 mOsmol kg^−1^; hyperosmolality ~ 420 mOsmol kg^−1^.

In the liquid phase, heat stress did not affect any of the alpha diversity parameters as expressed by ACE, Shannon and Simpson indices, and singles (*P* > 0.05, Table 3). Beta-diversity using weighted UniFrac and PCoA showed separation of clusters only in PC1 vs. PC3 plots due to heat stress (Figure 1(a)). Osmotic stress significantly enhanced Shannon and Simpson indices (*P* < 0.05, Table 3), and PCoA plots also displayed an effect of osmotic stress on archaeal community as indicated by a clear separation of clusters for all three principal components (Figure 1(b)). Supplementation of betaine did not show any effect on the beta-diversity of archaea (Figure 1(c)), but a significant betaine × osmolality interaction existed for all alpha diversity parameters (*P* < 0.05) except for ACE, which only showed a trend (*P* < 0.1, Table 3). Specifically, only the high dose of betaine resulted in a significant increase in ACE (*P* = 0.03), Simpson (*P* = 0.009), Shannon (*P* = 0.001), and singles indices (*P* = 0.009) during the osmotic stress condition and not during the normal osmolality condition (Figure 2).

**Figure 3:**
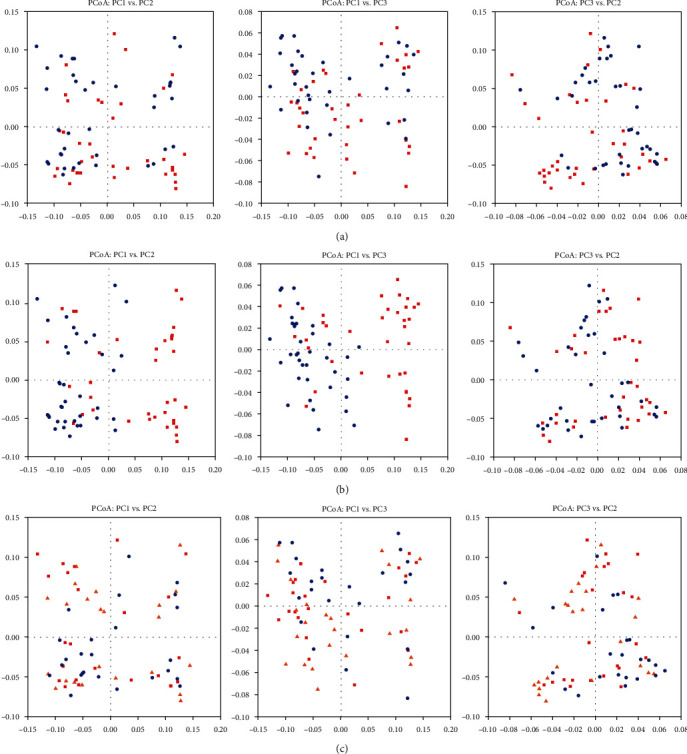
Principle coordinate analysis (PCoA) for beta-diversity of ruminal archaea in the solid phase of the incubation content of fermenters, under the effects of (a) temperature (red squares: 39.5°C; blue circles: 42°C), (b) osmolality^1^ (blue circles: normal; red squares: osmotic stress), and (c) betaine supplementation^2^ (red squares: control; orange triangles: low; blue circles: high). The first 3 components were plotted and in total principle component explained 77.61% of the total variation (PC1 = 47.78, PC2 = 21.46, and PC3 = 8.37%, respectively). ^1^Normal osmolality ~ 295 mOsmol kg^−1^; hyperosmolality ~ 420 mOsmol kg^−1^. ^2^Betaine levels: control (0), low (51), and high (286) ppm.

**Figure 4:**
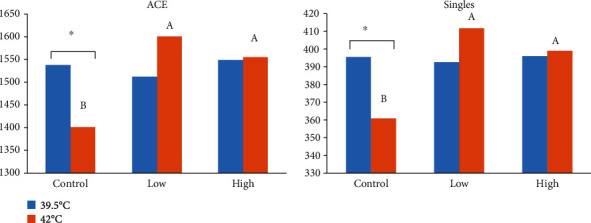
Diversity parameters of ruminal archaea in solid phase as affected by betaine addition^1^ and temperature. Different superscripts on heat stress (42°C) bars represent significant difference (*P* < 0.05). ^∗^*P* < 0.05. ^1^Betaine levels: control (0), low (51), and high (286) ppm.

In the solid phase, the effects of heat stress on archaea diversity were similar to those in the liquid phase. All alpha diversity parameters remained unaffected, and the separation of clusters in the PCoA plot was only prominent in the case of PC1 vs. PC3 (*P* > 0.05, Table 4, Figure 3(a)). Osmotic stress significantly lowered the diversity parameters ACE and singles (*P* < 0.05, Table 4), and the clustering pattern in the PCoA loading plot showed clear separation (Figure 3(b)). No change was observed in the alpha and beta-diversity parameters due to betaine supplementation at any level (*P* > 0.05, Table 4, Figure 3(c)). However, a trend of betaine × temperature interaction existed for ACE and singles (*P* < 0.1, Table 4), showing both betaine levels having an enhanced ACE and number of singles during the heat stress condition in the solid phase (Figure 4).

### 3.3. Archaea Composition as Affected by Incubation Conditions and Treatment

The sequencing of archaea amplicons resulted in quality 9,442,068 reads in 144 samples with a mean of 66,139 reads per sample. These reads could be clustered into 3016 unique OTUs with a minimum of 10 sequences per OTU. The evaluation was done with the nonnormalized data. Two phyla, *Crenarchaeota* (0.0008%–0.2%) and *Euryarchaeota* (99.8%–99.9%), were identified in both liquid and solid phases. At the genus level, Vadin CA11, *Methanosphaera*, *Methanosarcina*, *Methanobrevibacter*, *Methanobacterium*, *Methanimicrococcus*, and *Methanosaeta* were found in the liquid phase (Table 5). Except for the genus *Methanosaeta*, all other genera were also found in the solid phase (Table 6).

In the liquid phase, heat stress significantly promoted the genus *Methanosarcina* and showed a tendency to increase *Methanimicrococcus* at the expense of Vadin CA11 (Table 5). Osmotic stress significantly enhanced the abundance of phylum *Crenarchaeota*, and genera *Methanosphaera* and *Methanobacterium* (*P* < 0.05, Table 5). There was no betaine supplementation effect on the relative abundance of archaea at the genus and phylum levels (Table 5). However, a trend towards betaine × osmolality and betaine × osmolality × temperature interaction was seen for the genus *Methanimicrococcus* (*P* = 0.02) and *Methanosarcina* (*P* = 0.08), respectively. There was a significant negative correlation of the genus *Methanosphaera* with [2H] utilization and recovery based on the caproate propanyl CoA pathway, CH_4_ formation (per g OM and DM degraded), and [2H] utilization in CH_4_ (*P* < 0.05, Table 7). However, for genus *Methanobrevibacter*, a significant positive correlation was seen with CH_4_ formation corrected per g OM and DM degraded (*P* < 0.05, Table 7).

In the solid phase, heat stress showed a tendency to increase the relative abundance of phylum *Euryarchaeota* and genus *Methanosphaera* (*P* < 0.1, Table 6). The genus *Methanimicrococcus* was significantly increased at the expense of Vadin CA11 due to heat stress in comparison to normal rumen temperature conditions (*P* ≤ 0.05). Osmotic stress conditions significantly altered the relative abundance of *Methanobrevibacter* and *Methanimicrococcus*, with the former being increased at the expense of the latter (*P* < 0.05, Table 7). The abundance of phylum *Euryarchaeota* was reduced with high-dose supplementation of betaine (*P* = 0.04). However, this effect was not seen at the genus level. A significant negative correlation of genus *Methanobrevibacter* existed with CH_4_ formation (per g OM and DM degraded), [2H] utilization in relation to CH_4_, and overall utilization and recovery of [2H] (*P* < 0.05, Table 8). The genus *Methanimicrococcus* showed positive correlation with utilization and recovery of [2H] only when caproate production was associated with the propanyl CoA pathway (*P* < 0.05, Table 8).

## 4. Discussion

Archaea occupy many ecological niches in the ruminal ecosystem, and they primarily function to scavenge H_2_ to keep the rumen milieu favourable for microbial fermentation [32]. Rumen archaea can be categorized as hydrogenotrophic, aceticlastic, or methylotrophic based on the preferred H_2_ substrate of formate, acetate, and methylamines, respectively, the earlier being most abundant in the rumen [33]. This study focused on understanding the community diversity and population changes of archaea within either the liquid or solid rumen phase under controlled temperature and osmotic stress, with and without betaine supplementation.

In the absence of betaine, both hyperosmolality and heat lowered the population diversity of archaea, suggesting that archaea are sensitive to a range of physiochemical stress factors. Notably, the effects were robust during osmotic stress than heat stress conditions. Consequently, parameters associated with methanogenesis, including [2H] utilization in relation to CH_4_, and CH_4_ production, which are primarily the functions of archaea, were suppressed during osmotic stress conditions. Similar findings have been previously reported by Bennink et al. [34] who found 12.4% less CH_4_ production with salt-induced elevated osmolality in the rumen of wethers. The earlier studies did not focus on the archaea community in relation to high osmolality, but feeding high-grain diets, which elevates ruminal osmolality [35], have been shown to reduce CH_4_ production and formation [36, 37], which favour the possible reduction or changes in archaea community structure. The current modifications in archaea diversity due to heat stress were too limited to affect the gas production parameters, which indicates that the metabolic role of archaea was relatively sustained unlike during osmotic stress conditions. It was also supported by an *in vitro* study of Bhatta et al. [38] who documented no effect of mild heat stress on total fermentation gas and CH_4_ production. A strong effect of osmotic stress may be partly explained by a reduction of the substrate for methanogenesis as this stress condition also suppressed overall ruminal fermentation [3].

The ruminal population of archaea was not entirely resilient against stressors; nevertheless, this group of microbes was physiologically more stress tolerant than rumen bacteria; the higher sensitivity of ruminal bacteria to these stress factors has been reported in another study with similar incubation conditions [3].

Supplementation of high betaine dose counteracted and supported the diversity of free-floating archaea, which was depressed during osmotic stress conditions. It is reasonable to interpret that betaine supported archaea metabolism primarily through its osmolytic properties rather than being used as a substrate because CH_4_ production, a catabolic product of betaine degradation, did not show interaction between betaine and osmolality. The requirement of organic osmolytes like betaine considerably rises during osmotic stress [4], and that like other microbes, archaea also take up betaine during elevated osmolality due to salt gradients [39]. Betaine is a compatible organic solute and osmoprotective substance [1] which not only helps to maintain fluid balance but also prevents molecular disintegration during stressful conditions [4]. As already mentioned, the effect of heat stress on archaea diversity was less pronounced than that of osmotic stress, and even a low dose of betaine was enough to reverse the effects of increased temperature. This is in agreement with previous research that showed the thermoprotective role of betaine on microbial cells [6].

It is interesting that the beneficial effects of betaine during osmotic stress were not noticed in the solid phase archaea, which showed higher stability to osmotic stress. This is possibly due to the protection provided by the biofilm environment as these archaea are part of the ruminal biofilm [32]. *Methanimicrococcus* was the only genus sensitive to osmotic stress in the solid phase, which is in agreement with previous reports [40]. On the contrary, archaea in the liquid phase benefitted from betaine during osmotic stress as supported by increased diversity indices. Being highly water soluble [41] and dosed directly into the liquid phase, betaine seems to be readily available to archaea in the liquid phase, which might explain why it selectively promoted archaea in this phase. Furthermore, archaea are not the sole consumers of betaine, and the improved fermentation shown earlier [3] suggest that other microbes such as bacteria also utilized and benefitted from betaine.

Betaine supported archaeal diversity in the solid phase during heat stress, although it did not change the community structure at the genus level, which indicates that betaine is not required for function under low stress conditions. Nevertheless, our data indicate that Vadin CA11 is heat sensitive, whereas *Methanimicrococcus* can thrive under heat stress. The decrease in Vadin CA11 in response to heat was compensated by a concurrent increase in *Methanimicrococcus* which could be a result of competitive exclusion under physiochemical stress conditions, since both are methylotrophic [42, 43]. The current and previous results [3] of this experiment showed that betaine addition not only increased methanogenesis but also generally enhanced fermentation, so betaine might have supported ruminal microbes including archaea indirectly by being used as a compatible organic osmolyte.

In order to facilitate interspecies hydrogen transfer, archaea require a close association with rumen bacteria or protozoa. Protozoa species are more likely to be associated with the liquid phase, whereas rumen bacteria are most abundant in the solid phase [32]. Both liquid and solid phases differed in terms of diversity and composition of the archaea community structure. It would be reasonable to assume that such differences would be due to preferred interspecies interactions. Archaea were found to be more diverse in the solid phase than in the liquid phase as was also reported by Bowen et al. [44]. This is probably due to the higher metabolic activity in the biofilm, which leads to the higher concentration of available substrates for archaea accessing bacterial metabolites, compared to those associated with free-floating feed particles [45]. Furthermore, archaea are comparably slow-growing organisms; therefore, they require more time to reestablish in the liquid phase [44]. In the current study, *Methanobrevibacter*, Vadin CA11, and *Methanosphaera* showed the highest relative abundances, regardless of the digesta phase. However, there were differences in the correlations between the archaea genera and fermentation gas parameters between the digesta phases. In the liquid phase, *Methanobrevibacter* had positive correlation with [2H] utilized to form CH_4_, which is a more reliable estimator of archaea activity compared to CH_4_ formation itself, as CH_4_ is also a product of betaine degradation [46]. The stoichiometric production of CH_4_ is 1 mole per mole of betaine degradation [47]. Nevertheless, *Methanobrevibacter* also possessed a strong positive correlation with CH_4_ formation contrary to *Methanosphaera*, which was negatively correlated with CH_4_ formation in the liquid phase. The negative correlation between both of these genera with CH_4_ supports previous research *in vivo* [48]. However, *Methanobrevibacter* had a strong negative correlation with the formation of CH_4_ and [2H] incorporated into CH_4_ in the solid phase. Thus, members of *Methanobrevibacter* are likely to be active in scavenging H_2_ in the liquid phase, possibly due to syntrophic association with protozoa that provides a steady supply of the H_2_ substrate [49]. In comparison, solid phase members of *Methanobrevibacter* show less activity, which may be explained by a higher competition for H_2_ in the solid phase as members of this group are more diverse in the solid phase than in the liquid phase [44]. In an *in vivo* study, Danielsson et al. [50] did not find a correlation of CH_4_ with *Methanobrevibacter* and *Methanosphaera* at the genus level in the rumen of cows; however, at the species level, *Methanobrevibacter gottschalkii* manifested positive correlation with CH_4_ contrary to *Methanobrevibacter ruminantium*. However, in the current study, identification of the sequenced archaea to the species level was not possible for comparison of results. The taxonomic composition between solid and liquid phases differed only in terms of the genus *Methanosaeta*. As members of this genus are strictly aceticlastic, their exclusive presence in the liquid phase is likely due to acetate production in the liquid phase as a result of betaine degradation compared to the more well-described production of acetate in the digesta due to fibre degradation [42, 46, 51]. The current study shows that despite similar phylogeny, archaea groups have different roles in ruminal microenvironments and competition between these groups is dependent on substrate availability.

## 5. Conclusion

Members of *Methanobrevibacter*, Vadin CA11, and *Methanosphaera* genera were the most abundant taxa in both liquid and solid phases. Osmotic stress provided a more challenging environment to the fermentation and impacted the diversity and relative abundance of the archaea community as compared to heat stress. Archaea found in the liquid phase were less tolerant to osmotic stress than those in the solid phase. At the genus level, *Methanimicrococcus* in the solid phase and Vadin CA11 in both phases were highly sensitive to osmotic stress and heat stress, respectively. A high dose of betaine was able to reduce the detrimental effects of osmotic stress on archaea diversity in the liquid phase but not in the solid phase. However, even a low dose of betaine is enough to counteract the effects of heat stress on archaea diversity in the solid phase *in vitro*.

## Figures and Tables

**Table 1:** Stoichiometric production and utilization of metabolic [2H] during synthesis of major end products of fermentation.

End product	Stoichiometry equation	[2H] balance^∗^
Acetate (CH_3_COOH)	C_6_H_12_O_6_+2H_2_O1 ⟶ 2CH_3_COOH+4[2H]+2CO_2_	+2
Propionate (CH_3_CH_2_COOH)	C_6_H_12_O_6_+2[2H] ⟶ 2CH_3_CH_2_COOH+2H_2_O	-1
Butyrate (CH_3_(CH_2_)_2_COOH)	C_6_H_12_O_6_ ⟶ CH_3_(CH_2_)_2_COOH+2[2H]+2CO_2_	+2
Valerate (CH_3_(CH_2_)_3_COOH)	C_6_H_12_O_6_ +[2H] ⟶ CH_3_(CH_2_)_3_COOH+CO_2_+2H_2_O	-1
Caproate^a^ (CH_3_(CH_2_)_4_COOH)	C_6_H_12_O_6_+4[2H] ⟶ CH_3_(CH_2_)_4_COOH+4H_2_O	-4
Caproate^b^ (CH_3_(CH_2_)_4_COOH)	3C_6_H_12_O_6_ ⟶ 2CH_3_(CH_2_)_4_COOH+4[2H]+2H_2_O+6CO_2_	+2
Methane (CH_4_)	CO_2_+4[2H] ⟶ CH_4_+2H_2_O	-4

^a^Propanyl-CoA as intermediate. ^b^Acetyl-CoA as intermediate. ^∗^Moles [2H] utilized or produced per mole of the fermentation end product.

**Table 2:** Fermentation gas parameters and energy connected with methane.

Parameters	Osmolality^8^			Temperature (°C)			Betaine^9^				*P* value^$^		
	Normal	Hyper	SE^∗^	39.5	42	SE^∗^	Control	Low	High	SE^∗^	Osmo.	Temp.	Betaine
CH_4_ production (mL/d)	87.3	49.3	18.98	66.7	69.9	1.62	50.6^c^	60.1^b^	94.2^a^	13.23	<0.0001	0.2362	<0.0001
CO_2_ production (mL/d)	423.8	277.7	73.06	346.3	355.3	4.48	328.0^b^	341.3^b^	383.0^a^	16.58	<0.0001	0.4607	0.0013
Total fermentation gas^1^ (mL/d)	550.3	365.5	92.36	450.6	465.2	7.33	418.6^b^	438.2^b^	516.9^a^	30.05	<0.0001	0.3361	<0.0001
CH_4_ (% of total gas)	15.6	12.8	1.42	14.1	14.3	0.12	11.6^c^	13.2^b^	17.8^a^	1.87	<0.0001	0.432	<0.0001
CO_2_ (% of total gas)	77.2	76.0	0.63	76.9	76.3	0.28	77.8^a^	77.9^a^	74.1^b^	1.25	0.1106	0.474	<0.0001
Methane formation based on nutrient fermentation (mL/g degraded)													
CH_4_/g OM	13.5	7.9	2.82	10.7	10.7	0.00	7.8^c^	9.5^b^	14.8^a^	2.11	<0.0001	0.998	<0.0001
CH_4_/g DM	12.2	7.1	2.56	9.7	9.7	0.01	7.0^c^	8.6^b^	13.4^a^	1.91	<0.0001	0.976	<0.0001
MCR (%GE intake)^2^	1.63	0.92	0.354	1.24	1.30	0.03	0.94^c^	1.12^b^	1.75^a^	0.246	<0.0001	0.234	<0.0001
Metabolic hydrogen [2H] (mmol/d)													
Acetate produced	59.9	50.3	4.77	53.9	56.3	1.17	52.0^b^	55.5^a^	57.8^a^	1.68	<0.0001	0.058	0.0012
Butyrate produced^3^	20.8	21.3	0.26	21.1	20.9	0.09	20.5	21.1	21.5	0.30	0.4741	0.797	0.4898
Caproate produced	3.76	2.99	0.386	3.02	3.74	0.36	3.60	3.37	3.17	0.125	0.1171	0.142	0.7688
Propionate utilized	16.4	16.7	0.16	17.6	15.5	1.05	15.7	16.8	17.0	0.42	0.6826	0.008	0.314
Valerate utilized	3.23	2.75	0.241	2.77	3.20	0.21	3.01	3.01	2.94	0.023	0.0072	0.016	0.9305
Caproate utilized	7.5	6.0	0.77	6.0	7.5	0.72	7.2	6.7	6.3	0.25	0.1166	0.143	0.7679
CH_4_ utilized	13.8	7.8	3.00	10.5	11.1	0.26	8.0^c^	9.5^b^	14.9^a^	2.09	<0.0001	0.235	<0.0001
Metabolic hydrogen [2H] balance (mmol/d)													
Production (pathway 1)^4^	80.6	71.6	4.5	75.0	77.2	1.07	72.4	76.6	79.3	1.9	<0.0001	0.14	0.001
Utilization (pathway 1)	40.9	33.2	3.85	36.9	37.2	0.14	33.9	36.1	41.2	2.16	<0.0001	0.72	<0.0001
Gain (pathway 1)^6^	39.7	38.4	0.65	38.1	40.0	0.94	38.5	40.5	38.1	0.72	0.46	0.29	0.51
Recovery% (pathway 1)^7^	51.2	47.2	1.99	49.7	48.7	0.46	47.4	47.7	52.4	1.6	0.01	0.54	0.01
Production (pathway 2)^5^	84.4	74.6	4.89	78.0	80.9	1.43	76.0	79.9	82.4	1.86	<0.0001	0.06	0.003
Utilization (pathway 2)	33.4	27.2	3.08	30.9	29.7	0.57	26.7	29.4	34.8	2.40	<0.0001	0.10	<0.0001
Gain (pathway 2)^6^	51.0	47.4	1.81	47.1	51.1	2.01	49.3	50.6	47.6	0.86	0.04	0.02	0.40
Recovery% (pathway 2)^7^	39.9	36.8	1.53	39.8	36.9	1.43	35.3	37.2	42.5	2.15	0.01	0.02	<0.0001

^1^Total fermentation gas = CH_4_ + CO_2_ + O_2_. ^2^Methane conversion rate. ^3^Osmolality × temperature interaction. ^4^Propanyl CoA as intermediate in caproate formation. ^5^Acetyl CoA as intermediate in caproate formation. ^6^Production − utilization. ^7^Utilization/production × 100. ^8^Normal osmolality ~ 295 mOsmol kg^−1^; hyperosmolality ~ 420 mOsmol kg^−1^. ^9^Betaine levels: control (0), low (51), and high (286) ppm. ^∗^Standard error. ^$^*P* value tests the fixed effect of osmolality (*n* = 36), temperature (*n* = 36), and betaine addition (*n* = 24). Significance: *P* < 0.5.

**Table 3:** Measures of alpha diversity of ruminal archaea community in fermenters associated with the liquid phase as determined using QIIME and 16S rRNA sequences.

Estimators	Osmolality^1^			Temperature (°C)			Betaine^2^				*P* value^$^			
	Normal	Hyper	SE^∗^	39.5	42	SE^∗^	Control	Low	High	SE^∗^	*O* ^3^	*T* ^3^	*B* ^3^	Interaction^4^
ACE	1488.4	1485.1	52.3	1468.2	1505.3	52.32	1463.3	1495.8	1501.2	55.7	0.93	0.33	0.68	(*B* × *O*)
Shannon index	4.60	4.81	0.07	4.68	4.73	0.078	4.6672	4.7177	4.7426	0.08	<0.001	0.28	0.40	*B* × *O*, (*B* × *T*)
Simpson index	0.9031	0.9117	0.004	0.9083	0.9066	0.004	0.9045	0.9091	0.9087	0.004	0.003	0.55	0.35	*B* × *O*, *B* × *T*
Singles	385.42	381.15	13.3	378.76	387.81	13.38	375.38	385.34	389.13	14.23	0.66	0.35	0.49	*B* × *O*

^1^Normal osmolality ~ 295 mOsmol kg^−1^; hyperosmolality ~ 420 mOsmol kg^−1^. ^2^Betaine levels: control (0), low (51), and high (286) ppm. ^3^*O*: osmolality; *T*: temperature; *B*: betaine. ^4^Only effects with significance (*P* ≤ 0.05) or tendency marked with bracket (*P* ≤ 0.10) are listed. ^∗^Standard error. ^$^*P* value is for testing the fixed effect of osmolality (*n* = 36), temperature (*n* = 36), and betaine addition (*n* = 24). Significance: *P* < 0.05.

**Table 4:** Measures of alpha diversity of ruminal archaea community in fermenters associated with the solid phase as determined using QIIME and 16S rRNA sequences.

Estimators	Osmolality^1^			Temperature (°C)			Betaine^2^				*P* value^$^			
	Normal	Hyper	SE^∗^	39.5	42	SE^∗^	Control	Low	High	SE^∗^	*O* ^3^	*T* ^3^	*B* ^3^	Interaction^4^
ACE	1622.5	1430.2	32.8	1533.2	1519.5	32.9	1469	1557.3	1552.2	38.03	<0.001	0.71	0.11	(*B* × *T*)
Shannon index	4.83	4.794	0.04	4.84	4.77	0.04	4.74	4.87	4.82	0.05	0.50	0.23	0.19	
Simpson index	0.909	0.9093	0.002	0.911	0.907	0.002	0.903	0.912	0.911	0.003	0.94	0.29	0.11	
Singles	417.2	368.35	8.90	394.9	390.6	8.91	402.2	402.2	397.5	10.19	<0.001	0.66	0.12	(*B* × *T*)

^1^Normal osmolality ~ 295 mOsmol kg^−1^; hyperosmolality ~ 420 mOsmol kg^−1^. ^2^Betaine levels: control (0), low (51), and high (286) ppm. ^3^*O*: osmolality; *T*: temperature; *B*: betaine. ^4^Only effects with significance (*P* ≤ 0.05) or tendency marked with bracket (*P* ≤ 0.10) are listed. ^∗^Standard error. ^$^*P* value is for testing the fixed effect of osmolality (*n* = 36), temperature (*n* = 36), and betaine addition (*n* = 24). Significance: *P* < 0.05.

**Table 5:** Community structure of ruminal archaea in the liquid phase as affected by incubation conditions and betaine supplementation *in vitro.*

	Osmolality^1^			Temperature (°C)			Betaine^2^				*P* value^$^			
	Normal	Hyper	SE^∗^	39.5	42	SE^∗^	Control	Low	High	SE^∗^	*O* ^3^	*T* ^3^	*B* ^3^	Interaction^4^
Phyla														
*Crenarchaeota*	8 × 10^−6^	6 × 10^−5^	1.9 × 10^−5^	4 × 10^−5^	2 × 10^−5^	1.9 × 10^−5^	7 × 10^−5^	1 × 10^−5^	1 × 10^−5^	2.4 × 10^−5^	0.04	0.60	0.22	
*Euryarchaeota*	0.993	0.993	0.000	0.993	0.993	0.000	0.993	0.993	0.993	0.000	0.28	0.59	0.79	
Genera														
Vadin CA11	0.303	0.305	0.017	0.320	0.287	0.017	0.305	0.305	0.301	0.018	0.86	0.008	0.94	
*Methanosphaera*	0.043	0.069	0.014	0.052	0.060	0.014	0.059	0.057	0.052	0.015	0.04	0.53	0.89	
*Methanosarcina*	7 × 10^−7^	8 × 10^−7^	0.000	1 × 10^−6^	0.000	0.000	5 × 10^−7^	1 × 10^−6^	6 × 10^−7^	0.000	0.92	0.04	0.69	(*B* × *O* × *T*)
*Methanobrevibacter*	0.49	0.49	0.013	0.49	0.49	0.013	0.486	0.496	0.500	0.014	0.44	0.94	0.61	
*Methanobacterium*	1 × 10^−5^	2 × 10^−5^	5.4 × 10^−6^	1 × 10^−5^	2 × 10^−5^	5 × 10^−6^	2 × 10^−5^	2 × 10^−5^	1 × 10^−5^	6 × 10^−6^	0.03	0.42	0.38	
*Methanimicrococcus*	0.032	0.020	0.006	0.019	0.033	0.006	0.028	0.032	0.018	0.007	0.13	0.09	0.34	(*B* × *O*)
*Methanosaeta*	3 × 10^−7^	1 × 10^−10^	0.000	1 × 10^−10^	3 × 10^−7^	0.000	0.000	2 × 10^−10^	5 × 10^−7^	0.000	0.32	0.32	0.37	

^1^Normal osmolality ~ 295 mOsmol kg^−1^; hyperosmolality ~ 420 mOsmol kg^−1^. ^2^Betaine levels: control (0), low (51), and high (286) ppm. ^3^*O*: osmolality; *T*: temperature; *B*: betaine. ^4^Only effects with significance (*P* ≤ 0.05) or tendency marked with bracket (*P* ≤ 0.10) are listed. ^∗^Standard error. ^$^*P* value is for testing the fixed effect of osmolality (*n* = 36), temperature (*n* = 36), and betaine addition (*n* = 24). Significance: *P* < 0.05.

**Table 6:** Community structure of ruminal archaea in solid phase as affected by incubation conditions and betaine supplementation *in vitro.*

	Osmolality^1^			Temperature (°C)			Betaine^2^				*P* value			
	Normal	Hyper	SE^∗^	39.5	42	SE^∗^	Control	Low	High	SE^∗^	*O* ^3^	*T* ^3^	*B* ^3^	Interaction^4^
Phyla														
*Crenarchaeota*	0.001	0.001	0.0008	0.002	5 × 10^−4^	0.0008	5 × 10^−4^	0.001	0.002	0.001	0.98	0.10	0.26	
*Euryarchaeota*	0.99	0.99	0.0007	0.991	0.993	0.0007	0.994	0.993	0.991	0.001	0.19	0.07	0.09	
Genera														
Vadin CA11	0.27	0.26	0.016	0.293	0.2426	0.0162	0.288	0.253	0.260	0.018	0.56	0.001	0.15	
*Methanosphaera*	0.13	0.13	0.0308	0.115	0.159	0.030	0.119	0.0328	0.166	0.125	0.96	0.06	0.19	
*Methanosarcina*	2 × 10^−5^	2 × 10^−4^	1 × 10^−4^	2 × 10^−4^	1 × 10^−5^	1 × 10^−4^	1 × 10^−5^	2 × 10^−4^	9 × 10^−5^	1 × 10^−6^	0.29	0.20	0.46	
*Methanobrevibacter*	0.45	0.50	0.0208	0.494	0.463	0.0208	0.487	0.454	0.494	0.023	0.02	0.14	0.26	(*O* × *T*)
*Methanobacterium*	3 × 10^−5^	3 × 10^−5^	7 × 10^−6^	3 × 10^−5^	3 × 10^−5^	7 × 10^−6^	2 × 10^−5^	3 × 10^−5^	4 × 10^−5^	7 × 10^−6^	0.58	0.42	0.16	
*Methanimicrococcus*	0.01	0.003	0.002	0.0045	0.011	0.0025	0.006	0.005	0.011	0.003	0.02	0.05	0.33	(*O* × *T*)

^1^Normal osmolality ~ 295 mOsmol kg^−1^; hyperosmolality ~ 420 mOsmol kg^−1^. ^2^Betaine levels: control (0), low (51), and high (286) ppm. ^3^*O*: osmolality; *T*: temperature; *B*: betaine. ^4^Only effects with significance (*P* ≤ 0.05) or tendency marked with bracket (*P* ≤ 0.10) are listed. ^∗^Standard error. ^$^*P* value is for testing the fixed effect of osmolality (*n* = 36), temperature (*n* = 36), and betaine addition (*n* = 24). Significance: *P* < 0.05.

**Table 7:** Pearson's correlation coefficient analysis for archaea genera in liquid phase compared to fermentation parameters and gas production *in vitro.*

	*Methanimicrococcus*	*Methanobacterium*	*Methanobrevibacter*	*Methanosaeta*	*Methanosarcina*	*Methanosphaera*	Vadin CA11
Total fermentation gas	0.22	-0.08	0.18	0.11	-0.03	**-0.24**	-0.14
CH_4_	0.11	-0.06	0.18	0.13	-0.04	**-0.24**	-0.12
CO_2_	0.23	0.00	0.04	-0.04	0.06	-0.17	-0.07
CH_4_/g OM	-0.02	-0.03	**0.32**	0.14	-0.03	**-0.27**	-0.08
CH_4_/g DM	-0.02	-0.03	**0.32**	0.14	-0.03	**-0.26**	-0.09
MCR (%GE intake)	0.11	-0.06	0.18	0.13	-0.04	**-0.24**	-0.12
[2H] produced acetate	-0.08	0.01	-0.11	**0.31**	0.09	0.18	-0.16
[2H] produced butyrate	0.00	-0.13	0.16	-0.02	-0.11	-0.12	0.12
[2H] produced caproate	**0.26**	-0.04	-0.14	0.08	0.10	-0.18	0.10
[2H] produced (pathway 1)^1^	-0.08	-0.04	-0.04	**0.32**	0.05	0.14	-0.12
[2H] produced (pathway 2)^2^	-0.02	-0.05	-0.07	**0.33**	0.07	0.10	-0.09
[2H] utilized propionate	**-0.30**	-0.06	0.06	0.02	0.10	0.15	0.08
[2H] utilized valerate	0.21	0.09	-0.23	0.21	0.12	-0.06	0.12
[2H] utilized caproate	**0.26**	-0.04	-0.14	0.08	0.10	-0.18	0.10
[2H] utilized CH_4_	0.11	-0.05	0.18	0.12	-0.03	**-0.23**	-0.12
[2H] utilized (pathway 1)	0.10	-0.10	0.05	0.21	0.13	**-0.23**	0.05
[2H] utilized (pathway 2)	-0.09	-0.08	0.17	0.16	0.06	-0.11	-0.02
[2H] gain (pathway 1)	0.10	-0.11	0.06	0.21	0.14	**-0.23**	0.06
[2H] gain (pathway 2)	0.03	-0.006	-0.18	**0.27**	0.04	0.18	-0.09
[2H] recovery (pathway 1)	0.17	-0.07	0.05	-0.04	0.09	**-0.33**	0.15
[2H] recovery (pathway 2)	-0.08	-0.03	0.02	-0.06	0.002	-0.17	0.05

^∗^Bold cells show a significant correlation (*P* < 0.05). ^1^Propanyl CoA as intermediate in caproate formation. ^2^Acetyl CoA as intermediate in caproate formation.

**Table 8:** Pearson's correlation coefficient analysis for archaea genera in solid phase compared to fermentation parameters and gas production *in vitro.*

	*Methanimicrococcus*	*Methanobacterium*	*Methanobrevibacter*	*Methanosaeta*	*Methanosarcina*	*Methanosphaera*	Vadin CA11
Total fermentation gas	0.27	0.19	**-0.28**	-0.21	0.03	0.17	-0.08
CH_4_	0.22	**0.26**	**-0.29**	-0.18	-0.01	0.17	-0.08
CO_2_	0.06	-0.12	-0.02	0.03	0.00	0.07	-0.14
CH_4_/g OM	0.12	**0.30**	**-0.26**	-0.19	0.00	0.19	-0.03
CH_4_/g DM	0.11	**0.30**	**-0.26**	-0.19	0.00	0.19	-0.03
MCR (%GE intake)	0.22	**0.26**	**-0.29**	-0.18	-0.01	0.17	-0.08
[2H] produced acetate	-0.05	-0.05	-0.09	0.16	0.03	0.16	-0.08
[2H] produced butyrate	0.06	-0.10	0.18	-0.05	-0.08	-0.18	0.10
[2H] produced caproate	**0.30**	-0.08	-0.05	-0.09	-0.11	-0.12	0.01
[2H]^+^ produced (pathway 1)	-0.03	-0.09	-0.02	0.14	0.0002	0.09	-0.04
[2H] produced (pathway 2)	0.03	-0.11	-0.03	0.12	-0.02	0.06	-0.03
[2H] utilized propionate	**-0.26**	-0.07	0.10	0.09	0.02	0.03	0.02
[2H] utilized valerate	**0.23**	0.01	-0.20	-0.08	-0.16	0.11	-0.12
[2H] utilized caproate	**0.30**	-0.08	-0.05	-0.09	-0.11	-0.12	0.01
[2H] utilized CH_4_	0.21	**0.26**	**-0.29**	-0.18	0.00	0.17	-0.08
[2H] utilized (pathway 1)	**0.25**	0.11	**-0.24**	-0.16	-0.10	0.08	-0.06
[2H] utilized (pathway 2)	0.03	0.18	-0.22	-0.10	-0.02	0.19	-0.07
[2H] gain (pathway 1)	-0.19	-0.17	0.12	**0.25**	0.06	0.04	0.00
[2H] gain (pathway 2)	0.02	**-0.23**	0.09	0.20	-0.01	-0.04	0.00
[2H] recovery (pathway1)	**0.28**	0.15	-0.21	**-0.24**	-0.10	0.00	-0.05
[2H] recovery (pathway2)	0.04	**0.26**	-0.22	-0.18	-0.01	0.16	-0.05

^∗^Bold cells show a significant correlation (*P* < 0.05). ^1^Propanyl CoA as intermediate in caproate formation. ^2^Acetyl CoA as intermediate in caproate formation.

## Data Availability

Sequencing data are available with no restrictions in the BioProject SRA database under the accession number PRJNA602990.

## Supplementary Materials

Supplementary Materials Supplementary Figure 1: daily shift in pH for normal osmolality and hyperosmolality conditions.^1^
