# Supplementary material for: Betaine Modulates Rumen Archaeal Community and Functioning during Heat and Osmotic Stress Conditions In Vitro
Source: Archaea. 2020 Oct 22;2020:8875773. doi: 10.1155/2020/8875773 (PMC7599403; doi:10.1155/2020/8875773)
Supplement: Supplementary Materials — Supplementary Figure 1: daily shift in pH for normal osmolality and hyperosmolality conditions.1 [file 8875773.f1.docx]

**Supplementary figure 1: Daily shift in pH for normal osmolality and hyper osmolality conditions^1^.**


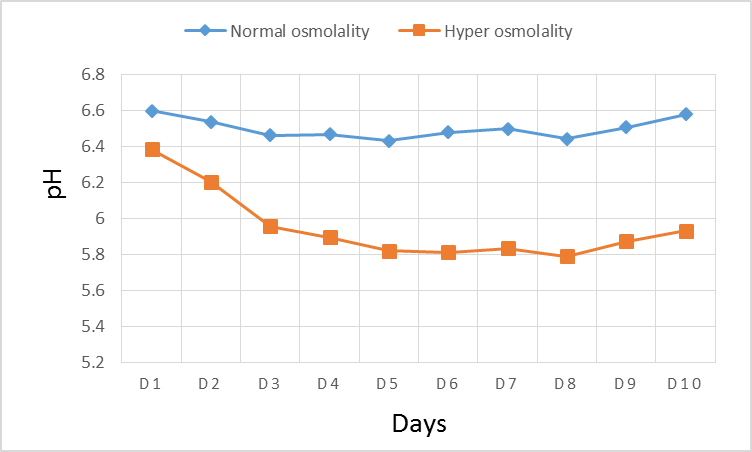


^1^Normal osmolality~295 mOsmol kg-1, Hyper osmolality~420 mOsmol kg-1
